# Integrating Bioinspired Natural Adhesion Mechanisms into Modified Polyacrylate Latex Pressure-Sensitive Adhesives

**DOI:** 10.3390/polym16172404

**Published:** 2024-08-24

**Authors:** Chunyuan Jiang, Xinrui Zhang, Xinyue Zhang, Xingjian Li, Shoufang Xu, Yinwen Li

**Affiliations:** 1 College of Chemistry and Chemical Engineering, Linyi University, Linyi 276000, China; jchunyuan1229@163.com (C.J.); zhangxinrui1011@163.com (X.Z.); xingjianli@lyu.edu.cn (X.L.); xshfang1981@163.com (S.X.); 2 College of Materials Science and Engineering, Linyi University, Linyi 276000, China; zhangxinyue0110@126.com

**Keywords:** modified polyacrylate latex PSAs (HPSAs), 3,4-dihydroxybenzaldehyde, adhesive strength, water resistance, adhesion mechanism

## Abstract

For polyacrylate latex pressure-sensitive adhesives (PSAs), high peel strength is of crucial significance. It is not only a key factor for ensuring the long-lasting and effective adhesive force of polyacrylate latex PSAs but also can significantly expand their application scope in many vital fields, such as packaging, electronics, and medical high-performance composite materials. High peel strength can guarantee that the products maintain stable and reliable adhesive performance under complex and variable environmental conditions. However, at present, the peel strength capacity of polyacrylate latex PSAs is conspicuously insufficient, making it difficult to fully meet the urgent market demand for high peel strength, and severely restricting their application in many cutting-edge fields. Therefore, based on previous experimental studies, and deeply inspired by the adhesion mechanism of natural marine mussels, in this study, a traditional polyacrylate latex PSA was ingeniously graft-modified with 3,4-dihydroxybenzaldehyde (DHBA) through the method of monomer-starved seeded semi-continuous emulsion polymerization, successfully synthesizing novel high-peel-strength polyacrylate latex pressure-sensitive adhesives (HPSAs) with outstanding strong adhesion properties, and the influence of DHBA content on the properties of the HPSAs was comprehensively studied. The research results indicated that the properties of the modified HPSAs were comprehensively enhanced. Regarding the water resistance of the adhesive film, the minimum water absorption rate was 4.33%. In terms of the heat resistance of the adhesive tape, it could withstand heat at 90 °C for 1 h without leaving residue upon tape peeling. Notably, the adhesive properties were significantly improved, and when the DHBA content reached 4.0%, the loop tack and 180° peel strength of HPSA_4_ significantly increased to 5.75 N and 825.4 gf/25 mm, respectively, which were 2.5 times and 2 times those of the unmodified PSA, respectively. Such superior adhesive performance of HPSAs, on the one hand, should be attributed to the introduction of the bonding functional monomer DHBA with a rich polyphenol structure; on the other hand, the acetal structure formed by the grafting reaction of DHBA with the PSA effectively enhanced the spatial network and crosslink density of the HPSAs. In summary, in this study, the natural biological adhesion phenomenon was ingeniously utilized to increase the peel strength of pressure-sensitive adhesives, providing a highly forward-looking and feasible direct strategy for the development of environmentally friendly polyacrylate latex pressure-sensitive adhesives.

## 1. Introduction

Biological adhesion phenomena in nature, distinguished by remarkable adhesive prowess, have come under intense scrutiny. Indeed, the robust adhesion properties of diverse species like marine barnacles, mussels, and abalones are attributed to their secretion of unique protein-based adhesives [1,2,3]. These adhesives are composed of various proteins, especially including a high content of the amino acid 3,4-dihydroxyphenylalanine (DOPA), which provides strong and flexible bonding and enables strong adhesion even in challenging marine environments. Therefore, the elucidation of these bioadhesive strategies in nature has been instrumental in offering invaluable insights into the genesis of advanced adhesive formulations for human applications.

Although these natural systems exhibit remarkable adhesive capabilities, most of the current commercial adhesives are petrochemical derivatives. Acrylic resin adhesives, as one of the most important adhesive branches, have received attention due to their excellent transparency, chemical stability, and consistently stable adhesion over a wide temperature range. Among them, polyacrylate latex pressure-sensitive adhesives (PSAs) have been widely applied in various fields, including medical devices, electronic products, and the automotive industry [4,5]. However, due to the emulsion polymerization process, involving emulsifiers, buffers, and functional monomers, large numbers of hydrophilic, polar, and ionic charge groups are introduced, correspondingly resulting in limited peel strength and water resistance of PSAs [6,7,8,9]. For instance, for pasting heavyweight decorative materials in construction engineering, fixing interior accessories in automotive manufacturing, making non-irritating medical patches [10,11,12], or adhering screens or components in electronic devices, acrylic pressure-sensitive adhesives with high peel strength are required, and at the same time, non-destructive peeling from the substrate must be achieved [13,14]. Nevertheless, the performance of current acrylic pressure-sensitive adhesives on the market is often unsatisfactory, and their peel strength needs to be enhanced.

Addressing this technological void, biomimicry, “emulating nature’s strategy”, has offered novel perspectives for the refinement of PSAs [15,16,17]. Bionic emulation of marine organisms’ adhesive mechanisms has been pivotal, particularly focusing on the mussel secretions that grace different material surfaces with robust and unscathed adhesion [18,19,20]. Research on such bionic mechanisms and in-depth discussion of adhesion principles have promoted the development of a new generation of adhesive materials [21,22,23,24]. For instance, since the broad investigation into catecholic moieties, their intrinsic robust adhesion, reactive nature, and crosslinking aptitude have been highlighted [25,26,27,28]. A groundbreaking study by White et al. [29] synthesized a pioneering tri-component ionic copolymer incorporating cationic, catechol, and phenyl groups, mimicking marine mussels’ adhesive proteins. In recent developments, Peng et al. [30] synthesized a cohesive hydrogel using a mixture of tannic acid and polyethylene glycol-b-polypropylene glycol-b-polyethylene glycol (F68), demonstrating instantaneous, robust, and reproducible adhesion underwater. The strong cohesive forces were attributed to hydrogen bonding between tannic acid and the polymer, as well as the hydrophobic core of F68 micelles. In another study, Zhan et al. [31] explored the impact of hydroxyl substitutions on the benzene ring pertaining to underwater adhesive strength. They synthesized various phenolic copolymers with phenol, catechol, and gallic acid tannin. The findings revealed that gallic acid tannin exhibited the strongest adhesive force, indicating that the tridentate structure offers enhanced binding capabilities at underwater interfaces. Through the studies conducted by researchers on novel adhesive materials, significant potential has been demonstrated by polyphenolic structures in the development of biomimetic adhesives, owing to their unique chemical properties and biocompatibility [18,32]. Although these modified adhesives have wide applications and cost-effectiveness, they also have significant drawbacks. Mainly, they exhibit environmental incompatibility, cumbersome decomposition processes, and suboptimal adhesion under adverse conditions such as humidity or high temperatures, contradicting the emerging ecological regulations advocating “green chemistry” and “clean production” [33,34].

**Figure 1:**
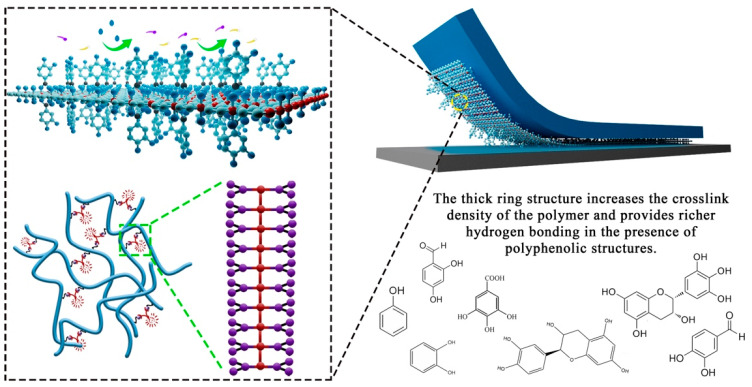
Adhesion mechanism of HPSAs.

Therefore, in this scheme, as shown in Figure 1, a bioinspired monomer, 3, 4-dihydroxybenzaldehyde (DHBA), was reacted with a traditional PSA, resulting in the fabrication of a series of high-peel-strength polyacrylate latex pressure-sensitive adhesives (HPSAs). This study explored the effects of the modified monomer on the preparation of HPSAs and their adhesive performance, as well as a series of performance characterizations. A comprehensive demonstration of a research example that combines the wisdom of natural adhesion systems with advanced material science is presented here, while also opening up a new perspective for the industrial production of eco-friendly water-based acrylic pressure-sensitive adhesives [35,36,37].

## 2. Experimental Section

### 2.1. Materials and Instruments

Acrylic acid (AA, 99%), butyl acrylate (BA, 99%), hydroxyethyl acrylate (HEA, 98%), methyl methacrylate (MMA, 99%), 2-ethylhexyl acrylate (EHA, 98%), and 3,4 -dihydroxybenzaldehyde (DHBA, 98%) were all purchased from Aladdin Reagents Co., Ltd., Shanghai, China. Ammonium nonylphenol polyoxyethylene ether sulfate (CO-436, industrial grade, Solvay) was purchased from Shanghai Sangjing Chemical Co., Ltd., Shanghai, China. Ammonium persulfate (APS), ammonia water (20%), sodium formaldehyde sulfoxylate, and tert-butyl hydrogen peroxide (all industrial grade) were all supplied by Linyi Pulima New Materials Co., Ltd., Shandong, China.

For measurement and analysis, the following equipment was employed: particle size and zeta potential instrument (ZetaPlus, Brookhaven, GA, USA); infrared spectroscopy (FT-IR, NEXUS 670, Nicolet, GA, USA); nuclear magnetic resonance (^1^H NMR, DMSO-*d6*, ECX 400, Bruker, Saarbrücken Germany); differential scanning calorimeter (DSC, Setline, Lyon, France); thermogravimetry (DSC-*T*_g_, Q600, TA Instruments, OH, USA); 120 KV transmission electron microscope (TEM, HT7800, HITACHI, TKY, Japan); constant-temperature and -humidity box (SHBY-40B, Haida, HenanChina).

### 2.2. Preparation of HPSAs

**Figure 2:**
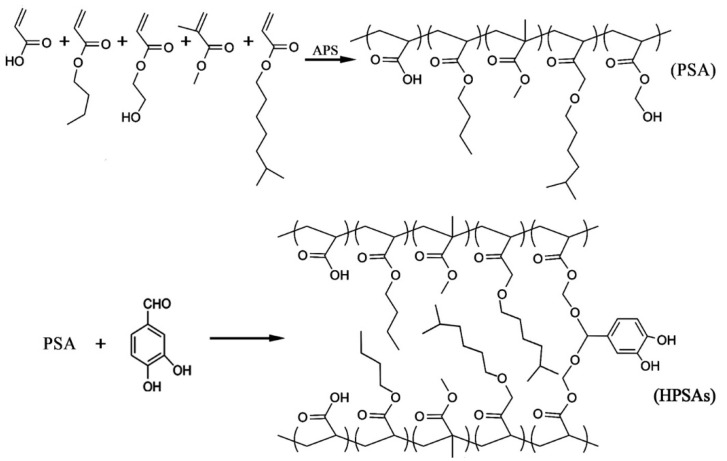
Synthesis routes of HPSAs.

The HPSAs were prepared by a pre-emulsification polymerization process. Taking HPSA_3_ as an example, the detailed synthesis steps were as follows: First, 2.3 g of CO436 and 60 g of deionized water were added to a pre-emulsification bottle and stirred until homogeneous. Then, a mixture of soft and hard monomers (AA, BA, HEA, and MMA) was added and emulsified at an appropriate speed for 50 min. Subsequently, 0.7 g of APS initiator was added, the mixture was stirred for 5–10 min, and the total pre-emulsification time was controlled at 60 min. Afterward, 150 g of deionized water and 0.7 g of CO436 were added to a reaction vessel and heated to 86 °C in a water bath. Then, 10% of the pre-emulsified emulsion and 0.2 g of APS initiator were added to the reaction vessel and maintained at this temperature for 30 min with minimal reflux. Later, the remaining pre-emulsified emulsion was added dropwise within 3 h. The reaction was continued at 86 °C for 1 h and then cooled to 70 °C to prepare the PSA. Then, DHBA was added, and the reaction was carried out for another 1 h. After the reaction was completed, the pH value was adjusted to 7–8 with ammonia water, and the mixture was filtered through a 180-mesh strainer to remove impurities, thus obtaining HPSAs. Figure 2 shows the synthesis routes of the HPSAs, and the formulations are listed in Table 1. For comparison before and after modification, the PSA was prepared using the same process parameters and formulation ratio as HPSA_1_, except that the functional monomer DHBA was not added.

### 2.3. Preparation of Pressure-Sensitive Adhesive Tape

**Figure 3:**
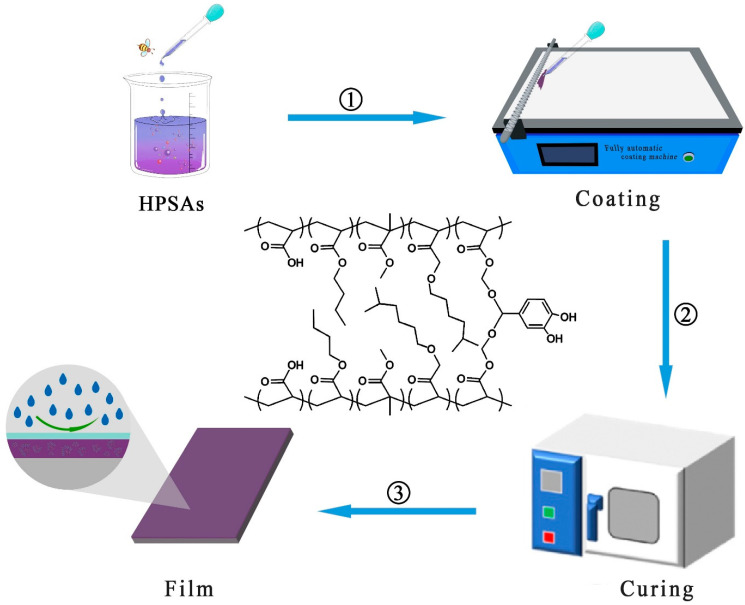
Preparation of pressure-sensitive adhesive tape.

The synthesized HPSAs were applied to a polyethylene (PE)-based black and white film (30 cm × 20 cm) to a specified thickness using a precision coating bar. An automatic film applicator was utilized to evenly spread the adhesive (coating weight: 15 g), as shown in Figure 3. Subsequently, the coated film was placed in a preheated drying oven at 75 °C for 15 min. Upon completion, the sample was removed and covered uniformly with a transparent polyethylene film on the corona-treated side to protect the adhesive layer. Lastly, the sample was returned to the drying oven at 75 °C for an additional hour of curing to produce the pressure-sensitive adhesive tape.

### 2.4. Performance Testing and Characterization

#### 2.4.1. Emulsion Viscosity and Structural Characterization

At 25 °C, with a 2# axis and at 60 rpm, the viscosity was measured using an NDJ-8S rotational viscometer. The average value of three parallel determinations was obtained for each measurement. The prepared polyacrylate emulsion was filtered through a 180-mesh filter, and its state, transparency, stratification during storage, and the static sedimentation of the emulsion were observed. Structural characterization was conducted by using Fourier-transform infrared spectroscopy (FT-IR) to measure the structure of the latex film. The samples were prepared by potassium bromide pellet pressing, and the test wavelength range was 400 to 4000 cm^−1^. The chemical structure and purity of the synthesized compounds were confirmed by using nuclear magnetic resonance (NMR) spectroscopy. The ^1^H NMR spectra were recorded at a frequency of 400 MHz on a Bruker AVANCE III 400 spectrometer. The solvent was deuterated dimethyl sulfoxide (DMSO-d6), and the internal standard was tetramethylsilane (TMS).

#### 2.4.2. Solid Content and Gel Fraction

The procedure for testing the solid content began with weighing a dry, clean weighing dish (m_0_, g), followed by adding a sample of HPSAs (m_1_, g), ideally between 1 and 2 g, to the dish. The dish was then placed in an oven at 110 °C for 2 h, followed by cooling in a vacuum oven to room temperature. The dish was weighed again (m_2_, g), and the solid content was calculated as (m_2_ − m_0_)/m_1_ × 100%. For gel fraction testing, after the reaction, the synthesized HPSAs were filtered through a 180-mesh screen, the gel collected on the screen was then dried in an oven at 110 °C for 1 h, subsequently weighed (m_1_, g), and the gel fraction was calculated as m_1_/m_0_ × 100%, where m_0_ represents the total mass of all monomers.

#### 2.4.3. Water Absorption and Water Resistance

The steps for testing the water absorption rate of HPSAs were as follows: HPSAs were poured into a kraft paper trough, and a film was formed at room temperature for 24 h. Then, it was dried in an oven at 40 °C for 96 h to obtain a dry HPSA film. Circular film samples with a diameter of approximately 5 cm were weighed (m_0_, g) and then immersed in water. Their masses (m_1_, g) were measured after soaking for 24 h, 48 h, and 72 h to calculate the water absorption rate as (m_1_ − m_0_)/m_0_ × 100%. The test steps for the water resistance of the pressure-sensitive adhesive tape were as follows: The pressure-sensitive adhesive tape was cut into film test strips with a length and width of 10 cm × 2.5 cm and placed in water. The alterations in the pressure-sensitive adhesive film layer were observed after soaking for 3 h, 6 h, 12 h, and 24 h.

#### 2.4.4. Emulsion Particle Size and Morphology

To test the particle size and its distribution, the prepared HPSAs were diluted 1000 times with deionized water in a quartz glass tube (allowing the light of a mobile phone to pass through) and stirred. The average particle size and dispersion index of the latex particles were measured at 25 °C using a zeta potential and particle size analyzer. Meanwhile, the diluted emulsion was dropped onto a copper grid coated with a Formvar film, stained with phosphotungstic acid, dried under an infrared lamp, and the morphology of the emulsion particles was observed using a transmission electron microscope (TEM).

#### 2.4.5. Differential Scanning Calorimetry (DSC)

The glass transition temperature of the PSA was determined using a DSC instrument. Under a nitrogen atmosphere, at a heating rate of 10 °C/min and a gas flow rate of 20 mL/min, samples (±10 mg) were heated from −60 °C to 80 °C, and the glass transition temperature (*T*_g_) was ascertained from the inflection point of the resultant endothermic flow curve.

#### 2.4.6. Thermogravimetry (TGA) and Hygrothermal Shock

The thermal stability and hygrothermal shock tests of the PSA and HPSAs were evaluated by TGA. About 10 milligrams of the sample was weighed in a standard alumina crucible and heated from 50 °C to 600 °C at a rate of 10 °C/min and a N_2_ flow rate of 100 mL/min. An accelerated aging test was carried out in a constant-temperature and -humidity curing box at 60 ± 2 °C and 90 ± 5% relative humidity for 96 h. Then, the tape (25 mm wide) was analyzed during the stripping process for the presence of residual adhesives.

#### 2.4.7. Adhesion Performance

The adhesion performance was characterized by loop tack, holding power, and 180° peel strength tests. Loop tack refers to the force required to separate a lightly pressed and quickly peeled adhesive tape from a substrate. The method used a 25 mm wide and 300 mm long tape, bent into an outward-facing loop, which was suspended from the clamps of a tensile machine. The tape was lowered at a rate of 300 mm/min until contact was made with a horizontal stainless steel plate, adhering by its own weight until an adhesive area of 25 × 25 mm^2^ was achieved. After a dwell time of 30 s, the tensile machine’s clamps were raised at 300 mm/min, and the peak force required to peel the tape was recorded in newtons (N). The 180° peel strength of the pressure-sensitive adhesive was determined in accordance with the GB/T 2792-2014; Test Method for Peel Strength of Adhesive Tapes. Standardization Administration of China: Beijing, China, 2014. Holding power was evaluated based on the GB/T 4851-1998; Test Method for Holding Power of Pressure Sensitive Adhesive Tapes. Standardization Administration of China: Beijing, China, 1998. Where a standard stainless steel panel coated with pressure-sensitive adhesive was hung vertically on the holding power tester with a 1 kg weight suspended, and the time for the sample to detach was recorded.

## 3. Results and Discussion

### 3.1. Synthesis and Physical Characterization of HPSAs

The preparation process of high-peel-strength polyacrylate latex pressure-sensitive adhesives (HPSAs) comprised two steps: The first step involved the pre-emulsification seed emulsion polymerization technique. MMA was used as the hard monomer, BA and EHA as soft monomers, and HEA and AA as the functional monomers to formulate polyacrylate pressure-sensitive adhesives (PSAs). The second step included the graft modification reaction, in which the hydroxyl functional groups in the PSA structure reacted with DHBA to generate HPSAs with polyphenol groups. The structure of the HPSAs was characterized by FT-IR and ^1^H NMR, and the results are shown in Figures S1 and S2, respectively. For the ^1^H NMR spectra, unfortunately, due to the large numbers of repetitive units (–COOCH_2_–) introduced from acrylic monomers (BA, MMA, AA, HEA, and EHA) in the HPSAs, the other peaks were very small. However, the characteristic peaks of DHBA (δ 9.36, 7.0–7.75) still confirmed that DHBA was indeed modified by the PSAs. Additionally, the following adhesion and water resistance properties also provided more accurate data to elucidate the synthesis of HPSAs.

The basic physical properties of the HPSAs, such as viscosity, stability, solid content, and gel fraction, were measured and characterized. The results are presented in Table 2. The viscosity results of the HPSAs revealed that the viscosity of the HPSAs initially decreased with the increase in the dosage of the DHBA functional monomer. It can be observed from Table 2 that the solid contents of HPSA_1_, HPSA_2_, HPSA_3_, HPSA_4_, and HPSA_5_ ranged from 48.3% to 50.1%, closely agreeing with the theoretical solid content (50%) within the allowable error range [37]. This strongly demonstrated that all polymerizable monomers participated in the emulsion polymerization reaction. However, due to the hydrophilicity of the modified monomer DHBA, as its content increased, soluble macromolecules formed in water could adsorb onto the surface of the latex particles, causing instability and gelation in the emulsion. Additionally, after six months of storage, no phase separation, precipitation, flocculation, viscosity changes, or abnormal morphology occurred. This indicates that a polyphenol aldehyde structure similar to a biological adhesive structure was formed in the HPSAs’ system, enhancing the intermolecular force and internal stability of the emulsion system.

### 3.2. Water Absorption and Water Resistance of HPSAs

**Figure 4:**
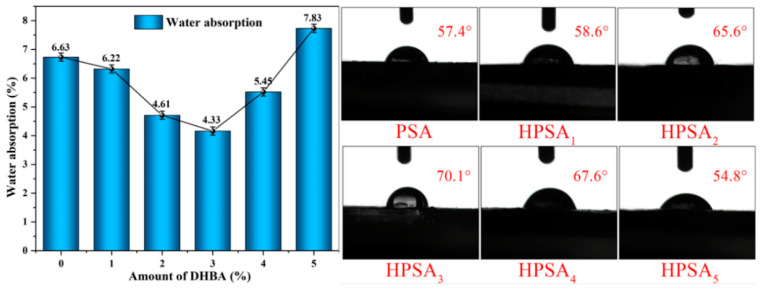
The water absorption rate and contact angle of HPSA films with different DHBA contents.

**Figure 5:**
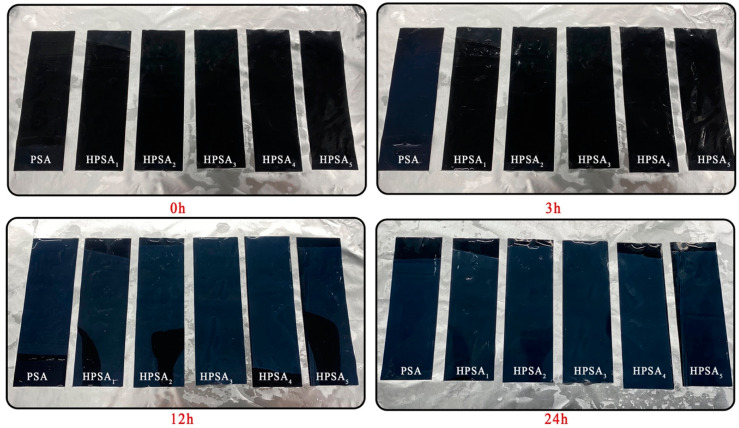
The soaking results of HPSA tapes with different DHBA contents.

Water absorption and water resistance both play crucial roles for PSAs. High water absorption is typically undesirable, as it adversely affects the adhesive strength and durability of the adhesive. In contrast, good water resistance implies that the pressure-sensitive adhesive can maintain its adhesiveness and persistence in moist or watery environments [10,11]. Therefore, this study explored the influence of the dosage of DHBA on the water absorption rate of HPSAs. The results showed that, with the increase in the dosage of the DHBA monomer, the water absorption rate of the HPSAs initially decreased and then increased. When the monomer dosage reached 3.0%, HPSA_3_ presented the best water absorption rate of 4.33%, while a further increase in the DHBA content led to an increase in water absorption. This can be attributed to the fact that a moderate acetal structure enhances the intermolecular crosslinking, forming a larger crosslinked molecular network. This appropriate increase in crosslinking density makes the crosslinked network structure more compact, reduces intermolecular friction, and thereby reduces water absorption [38]. However, the continuous increase in the DHBA content results in excessive crosslinking, making the polymer network overly tight and reducing the flexibility of the polymer. Additionally, an excess of unreacted DHBA may form hydrophilic regions on the polymer surface, increasing the hydrophilicity of the polymer and thereby raising the water absorption rate of the pressure-sensitive adhesive [39]. The contact angle of the latex film is depicted in Figure 4. Regarding the water resistance whiteness of the tape, the prepared tape was immersed in deionized water, and the color changes of the adhesive film layer were recorded after 3 h, 6 h, 12 h, and 24 h. Generally, the whitening of pressure-sensitive tapes is caused by water-induced swelling. Observing the tapes during these time periods, the PSA films began to show signs of whitening after 3 h of immersion, while the HPSAs did not exhibit this phenomenon until 12 h, after which whitening occurred. This indicates that the introduction of DHBA, inspired by the adhesion systems of marine organisms, can strengthen the crosslinked polymer network and inhibit the entry of water molecules into the acrylic film of the tape, thereby enhancing the water resistance whiteness of the tape. The experimental results are presented in Figure 5.

### 3.3. Particle Size and Morphology Analysis of HPSAs

To investigate the characteristics of the HPSAs further, the particle size and morphology of HPSAs with different DHBA contents were analyzed. The particle size and distribution of the polyacrylic latex PSAs have a significant impact on the adhesive properties. Extreme size deviations or uneven particle size distributions can severely compromise the wetting and adsorption effects of PSAs. This, in turn, influences the adhesive strength, cohesion, 180° peel strength, density, and water resistance of the film.

**Figure 6:**
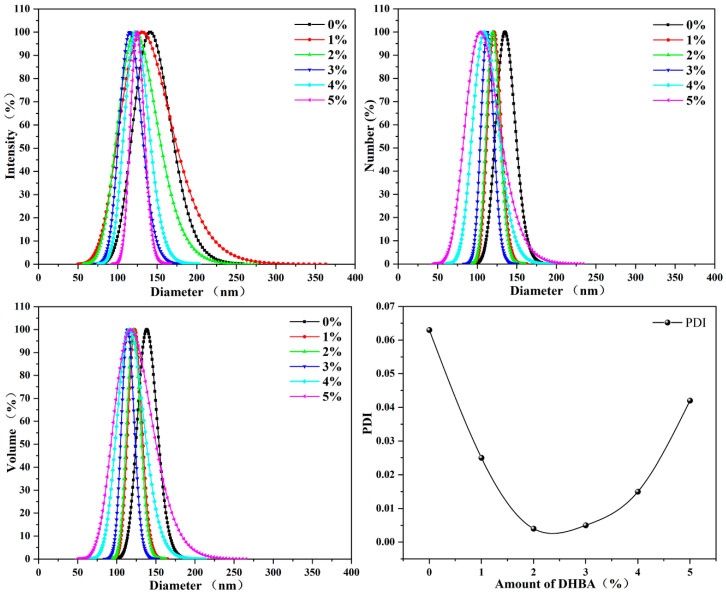
Particle size and distribution of HPSAs with different DHBA contents.

Since the solid content of high-peel-strength composite adhesives is typically controlled at approximately 50%, the solid content of the PSA and HPSAs prepared in this paper was also designed to be controlled within the range of 50 ± 1%. Due to the mutual adhesion among emulsion particles, direct measurement of the particle size and distribution may lead to reduced accuracy. Hence, the dynamic light scattering (DLS) method was employed to explore the particle size and distribution of HPSAs, with the emulsion diluted 1000 times. As shown in Figure 6, the average volume particle size of HPSAs is typically within the range of 114.0–139.8 nm—specifically, 122.9 nm, 121.3 nm, 114.0 nm, 116.2 nm, and 131.1 nm for HPSA_1_, HPSA_2_, HPSA_3_, HPSA_4_, and HPSA_5_, respectively. Additionally, the distribution range is narrow (PDI < 0.2). After introducing DHBA into the copolymer system, the emulsion particle size decreased, and the average volume particle size of PSA was 139.8 nm. This is mainly because the acetal reaction (a typical nucleophilic addition reaction) forms more crosslinking points between chains by utilizing the acetal structure, increasing the density of the polymer network. A denser network structure restricts the growth of polymer particles, thereby causing the particle size to decrease. Another possible reason is that the formation of the acetal structure can enhance the stability of polymer particles in the dispersion medium. The enhanced compatibility and stability can inhibit particle aggregation and make it difficult for larger particles [36]. Furthermore, as shown in Figure S3, HPSA_5_ exhibits a uniform spherical morphology with a particle size of approximately 70 nm, slightly smaller than the DLS result. This difference is due to the fact that TEM characterization depicts the conformation of dry latex particles, which are in a contracted state, ultimately resulting in a smaller size than that of the DLS result.

### 3.4. Glass Transition Temperature Analysis of HPSAs

**Figure 7:**
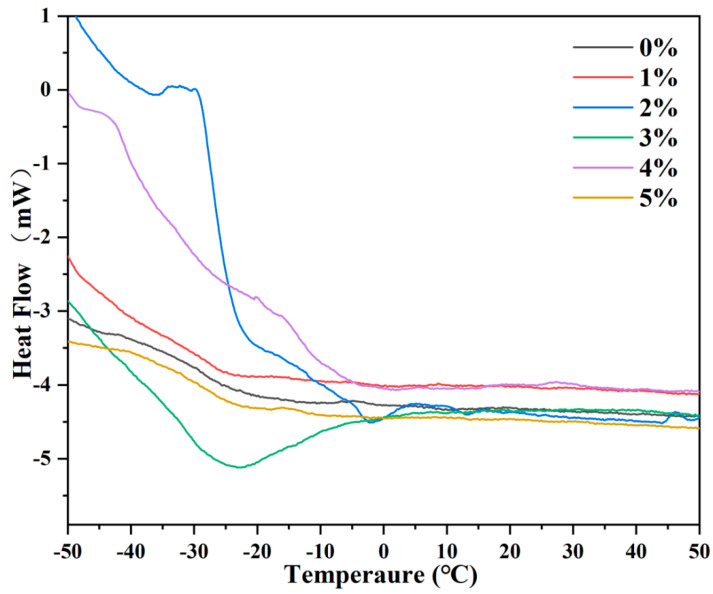
The *T*_g_ of HPSAs under different DHBA contents.

The glass transition temperature (*T*_g_) was a major factor considered during the formulation design process, representing the lowest temperature at which polymer segments could move [10,11,12]. This was because *T*_g_ significantly influenced the final bonding performance of PSAs, including initial tack, shear strength, and peel strength. The design process was guided by the FOX equation, and the actual *T*_g_ of HPSAs with different DHBA contents was measured using differential scanning calorimetry (DSC). The corresponding DSC curves are shown in Figure 7, and the detailed *T*_g_ results are summarized in Table 3. The *T*_g_ of PSA was affected by factors such as molecular weight, gel content, chain structure, and the interaction between copolymers and fillers. It can be clearly observed from Table 3 that with the increase in DHBA content, the *T*_g_ of HPSAs first decreased, then increased, and eventually approached the level of the PSA. The initial decrease in *T*_g_ was attributed to the plasticizing effect of a small amount of DHBA [32]. Taking HPSA_1_ as an example, the *T*_g_ of HPSA_1_ was only −39.31 °C, significantly lower than that of the PSA; the hydroxyl groups in its chemical structure enhanced the intermolecular interaction during the copolymerization process with BA and other monomers, thereby increasing the fluidity and flexibility of the polymer chains. However, with the continuous increase in DHBA content, the dihydroxy structure led to more crosslinking reactions, increasing the rigidity of the polymer and restricting the movement of the polymer chains, thereby increasing the *T*_g_. For HPSA_5_, with a 5.0% DHBA content, the *T*_g_ was −29.26 °C.

### 3.5. Thermogravimetric Analysis of HPSAs

**Figure 8:**
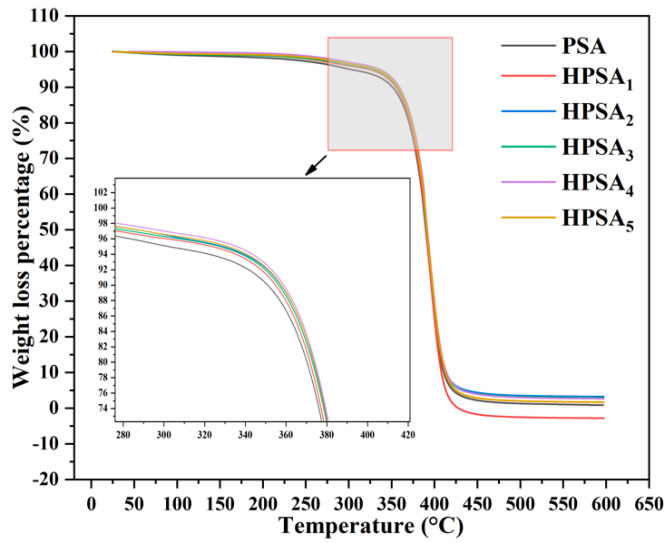
The heat resistance of HPSAs under different DHBA contents.

Thermogravimetric analysis (TGA) is a characterization method that is frequently used in scientific research to explore the thermal stability of materials. In this study, three parameters of TGA were adopted to evaluate the thermal stability of the modified high-peel-strength polyacrylate latex pressure-sensitive adhesives: the initial degradation temperature, the temperature at 10% mass loss (*T_loss10%_*), and the temperature at the maximum mass loss (*T_lossmax_*). The influence of DHBA content on the thermal stability of the HPSAs is shown in Figure 8. For instance, the thermal decomposition of the modified HPSA_4_ latex film began at 280 °C, which was significantly higher than that of the PSA latex film (210 °C), indicating that the heat resistance of the modified HPSAs was enhanced. This was attributed to the grafting of DHBA onto the PSA to form an acetal structure. It increased the rigidity of the polymer chains, limited the movement of the chains, and enhanced the crosslinking density between molecules, enabling the polymer to maintain its physical thermal stability at high temperatures. But interestingly, when the DHBA content increased to a certain extent, the heat resistance slightly decreased (HPSA_5_, 260 °C). This was because, as the DHBA content increased, it led to excessive crosslinking of the polymer network, making it hard and brittle and causing it to be more prone to fracture and decomposition when heated, thereby reducing the heat resistance [21].

**Figure 9:**
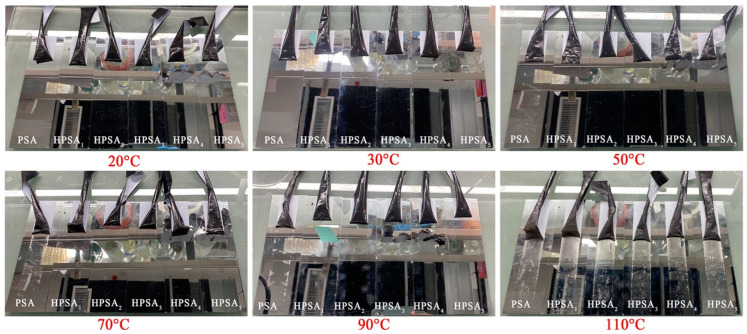
The influence of HPSAs with different contents of DHBA on the residual glue situation after 1 h of high-temperature treatment.

Furthermore, apart from investigating the thermal stability of the PSA at the molecular level using TGA, a more practical evaluation was carried out through high-temperature adhesion testing to assess the overall thermal stability of the HPSAs. In this test, the fabricated tape samples were adhered to mirror panels and were placed in ovens at different temperatures for a specific duration, and then they were cooled to room temperature. Eventually, the tapes were slowly peeled off (at 180°), and the presence of residual adhesive on the mirror panel was observed as an indicator of heat resistance. Figure 9 shows the effects of DHBA content on the residual adhesive of HPSAs after 1 h of high-temperature treatment, as summarized in Table 4. As indicated in the table, the acrylic PSA without any DHBA could only tolerate a temperature of 50 °C. Nevertheless, when the DHBA content was 4.0%, HPSA_4_ could withstand temperatures as high as 90 °C without any residual adhesive, significantly enhancing the thermal stability. Thus, the findings were consistent with the previously measured TGA data.

**Figure 10:**
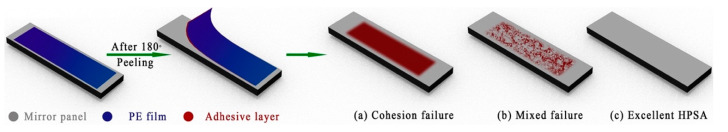
Residual situation of adhesive tape after peeling from stainless steel.

Based on the foregoing experiments, a damp heat-shock test was conducted to verify the durability of the modified tapes and accelerate their aging. The fabricated acrylic pressure-sensitive tapes were adhered to the mirror plates and placed in a constant-temperature and -humidity chamber under the accelerated aging conditions of 60 ± 2 °C and 90 ± 5% relative humidity for 96 h. After the experiment ended, the mirror plates were taken out and cooled to room temperature for 180° peeling. The quality of the tapes after peeling was evaluated, as shown in Figure 10. This experiment was designed to further validate the durability of the modified acrylic pressure-sensitive adhesives. Video S1 in the Supplementary Materials indicates the experimental results. There was no residue of the adhesive on the mirror plates with the HPSA tapes, fully demonstrating the durability of this material.

### 3.6. Adhesive Performance of HPSAs

To broaden the application scope of polyacrylate latex pressure-sensitive adhesives (PSAs), they are obliged to possess exceptional adhesive performance. PSAs were fabricated through emulsion polymerization. While the other conditions remained unchanged, 3,4-dihydroxybenzaldehyde (DHBA) was introduced. This process draws inspiration from nature and enhances the material performance through biomimetic approaches [36,40]. This study discusses the impact of the dosage of DHBA on the viscosity and adhesive performance of PSA emulsions before and after modification.

The viscosity results of the HPSAs, as shown in Table 2, indicated that the viscosity of the HPSAs decreased with the increase in DHBA content. For example, when the DHBA content was 5.0%, the viscosity of HPSA_5_ was 102.2 mPa.s. This phenomenon occurred because DHNA’s benzene ring and acetal structure, formed during polymerization, led to intermolecular crosslinking and larger molecular structures. However, this larger molecular structure could reduce the intermolecular friction, thereby reducing the viscosity [23]. But the viscosities of the PSA (145.7 mPa.s) and HPSAs all remained below 200 mPa.s, meeting the coating requirements, because when the viscosity was too high, it would affect the subsequent coating performance, resulting in an increase in the amount of adhesive, stripes and pinholes in the coating, etc. Therefore, the viscosity usually needed to be controlled below 200 mPa.s. Obviously, the content of DHBA had a very small influence on the viscosity of the PSAs.

**Figure 11:**
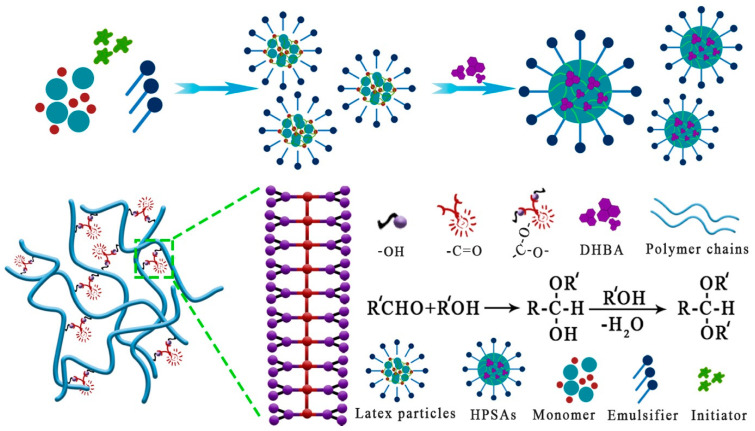
The adhesion mechanisms of HPSAs.

As shown in Figure 11, the introduction of DHBA with polyphenol groups to simulate the biological adhesion system significantly improved the peel strength of waterborne acrylic pressure-sensitive adhesives. The adhesion mechanism was that the formed acetal structure promoted the formation of more hydrogen bonds between polymer chains, contributing additional physical interactions, which greatly enhanced the material’s peel resistance and increased the crosslinking density, strengthening the rigidity and stability of the polymer network [18,41,42]. Moreover, the acetal structure provided a certain flexibility, enabling the stress during adhesion to be widely distributed across the contact surface, reducing the stress concentration and effectively preventing damage to the substrate surface. This balanced design of rigidity and flexibility demonstrated excellent adhesive capacity and stability [43,44,45].

**Figure 12:**
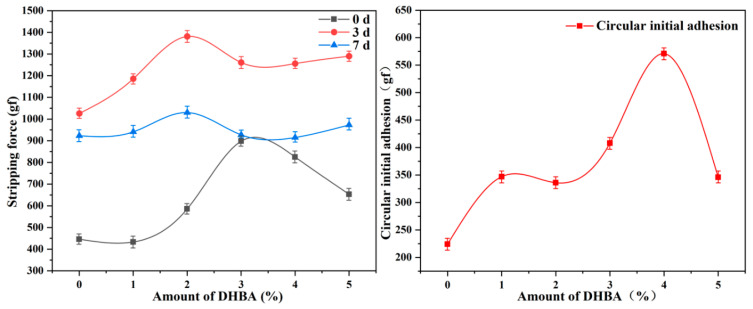
The influence of different contents of DHBA on the initial adhesion force and peel strength of HPSAs.

Therefore, the change in DHBA content was tested to observe its influence on the loop tack, 180° peel strength, and holding power of the HPSAs, and the results are presented in Table 5. As the DHBA content increased, the loop tack and 180° peel strength of the HPSAs initially showed an upward trend and then decreased. This was because a moderate increase in the DHBA content enhanced the crosslinking degree of the pressure-sensitive adhesive, increasing the interaction force between polymer chains, thereby improving tackiness and adhesion, which increased the loop tack force. However, excessive DHBA led to an overly rigid polymer network, reducing the flexibility of the polymer [46,47]. Moreover, high concentrations of DHBA might cause partial self-crosslinking, reducing the effective reaction with HEA, thus reducing the loop tack and 180° peel strength [48]. The experimental results indicated that when the DHBA content was 4.0%, HPSA_4_ achieved the best performance in terms of loop tack force (5.75 N), 180° peel strength (825.4 gf), and holding power (>72 h), as shown in Figure 12.

## 4. Conclusions

In conclusion, inspired by the natural biomimetic adhesion mechanism, through the utilization of 3,4-dihydroxybenzaldehyde (DHBA) with a polyphenol structure, a novel type of polyacrylate latex pressure-sensitive adhesive (HPSA) with high peel strength was successfully designed and obtained. The characteristics of this design are simple operation, controllable reaction conditions, and controllable particle size and distribution, all of which contribute to improving the stability of HPSAs. The superior adhesive performance of HPSAs can be attributed to the introduction of the polyphenol groups of DHBA itself, which have significant bonding functions, promoting the formation of more hydrogen bonds between polymer chains, contributing additional physical interactions, and greatly enhancing the material’s peel resistance, along with the formation of the acetal structure increasing the crosslinking density and the rigidity and stability of the polymer network. Additionally, the acetal structure provides a certain flexibility, enabling the stress generated during the adhesive process to be widely distributed on the contact surface, reducing the stress concentration and effectively preventing damage to the substrate surface. This balanced design of rigidity and flexibility demonstrates excellent bonding capacity and stability. This adhesion mechanism fully simulates the biological adhesion system, endowing HPSAs with excellent comprehensive performance. Therefore, when the content of DHBA was 4.0%, the annular initial adhesion force and 180° peel strength of HPSA_5_ were significantly increased to 5.75 N and 825.4 gf/25 mm, respectively, reaching 2.5 times and 2 times those of traditional PSAs, respectively. Meanwhile, its maximum heat resistance could reach 90 °C, and there was no residual adhesive. After conducting a 72 h water resistance test on the coating film, the optimal water absorption rate was 5.45%. Furthermore, the results of the 96 h damp heat-shock accelerated aging test conducted in a constant-temperature and -humidity chamber at 60 ± 2 °C and 90 ± 5% relative humidity show that there was no residual adhesive on the mirror plate. The experimental results indicate that, compared with traditional PSAs, HPSAs have significant improvements in peel strength, heat resistance, and water resistance. This study provides a feasible strategy for optimizing the performance of waterborne acrylic resins and represents the current development trend of environmentally friendly acrylic resins.

## Figures and Tables

**Table 1:** Formulae of HPSAs.

Sample	Monomer (g)					DHBA (%)
	MMA	BA	AA	HEA	EHA	
HPSA_1_	35	120	3	6	40	1
HPSA_2_	35	120	3	6	40	2
HPSA_3_	35	120	3	6	40	3
HPSA_4_	35	120	3	6	40	4
HPSA_5_	35	120	3	6	40	5
PSA	35	120	3	6	40	0

**Table 2:** Physical properties of HPSAs.

Sample	Stability (Six Months)	Viscosity (mPa.s)	Solid Content (%)	Gel Content (%)
HPSA_1_	No layering, unchanged viscosity	144.2	50.1	0
HPSA_2_	No layering, unchanged viscosity	116.4	49.2	0
HPSA_3_	No layering, unchanged viscosity	117.5	49.4	0
HPSA_4_	No layering, unchanged viscosity	114.5	48.9	0.13
HPSA_5_	No layering, unchanged viscosity	102.2	48.3	0.21
PSA	No layering, unchanged viscosity	145.7	49.6	0

**Table 3:** The *T*_g_ of HPSAs with different DHBA contents.

Sample	*T*_g_ Value (°C)	
	Theoretical (FOX)	Measured (DSC)
HPSA_1_	-	−39.31
HPSA_2_	-	−35.26
HPSA_3_	-	−31.41
HPSA_4_	-	−27.13
HPSA_5_	-	−29.26
PSA	−26.02	−28.57

**Table 4:** The influence of DHBA on the residual situation of HPSAs.

Sample		Residual Situation (1h)				
	20 °C	30 °C	50 °C	70 °C	90 °C	110 °C
HPSA_1_		-	-	Residue	Residue	Residue
HPSA_2_		-		Residue	Residue	Residue
HPSA_3_		-	-	-	Residue	Residue
HPSA_4_		-	-	-	-	Residue
HPSA_5_		-	-	-	-	Residue
PSA		-	Residue	Residue	Residues	Residues

**Table 5:** Effects of DHBA on adhesion properties of HPSAs.

Sample	180° Peel Strength (gf)			Ring Primary Adhesion (N)	Holding Force
	Initial	3 d	7 d		
HPSA_1_	433.5	1186.1	941.7	3.41	≈10 h
HPSA_2_	586.6	1381.3	1030.3	3.34	>72 h
HPSA_3_	898.3	1261.5	927.2	4.16	>72 h
HPSA_4_	825.4	1256.7	915.1	5.75	>72 h
HPSA_5_	653.3	1290.2	973.3	3.53	>72 h
PSA	446.4	1026.4	923.5	2.21	≈8 h

## Data Availability

Data are contained within the article and Supplementary Materials.

## Supplementary Materials

The following supporting information can be downloaded at https://www.mdpi.com/article/10.3390/polym16172404/s1, Figure S1: Infrared and visible spectra of PSA and HPSA_5_; Figure S2: ^1^H nuclear magnetic resonance spectra of PSA and HPSA_1_; Figure S3: Transmission image of HPSA_5_; Video S1: Results of damp heat-shock test.
