# Supplementary material for: Integrating Bioinspired Natural Adhesion Mechanisms into Modified Polyacrylate Latex Pressure-Sensitive Adhesives
Source: Polymers (Basel). 2024 Aug 24;16(17):2404. doi: 10.3390/polym16172404 (PMC11397013; doi:10.3390/polym16172404)
Supplement: Supplementary file 1 [file polymers-16-02404-s001.zip › polymers-3148078-supplementary.pdf]

Integrating Bioinspired Natural Adhesion Mechanisms into Modified Polyacrylate Latex Pressure-Sensitive Adhesives

Chunyuan Jiang [1,2], Xinrui Zhang [1,2], Xinyue Zhang [2], Xingjian Li [1,2], Shoufang Xu [1,2] and Yinwen Li [1,2,*]

[1] College of Chemistry and Chemical Engineering, Linyi University, Linyi 276000, China; jchunyuan1229@163.com (C.J.); zhangxinrui1011@163.com (X.Z.); xingjianli@lyu.edu.cn (X.L.); xshfang1981@163.com (S.X.)

[2] College of Materials Science and Engineering, Linyi University, Linyi 276000, China; zhangxinyue0110@126.com

* Correspondence: liyinwen06@126.com

FT-IR is a sensitive technique for analyzing the functional groups on the surface of materials. Through FTIR analysis, we further corroborated and dissected the alterations in functional groups during the reaction process. As shown in Figure S1, the spectral comparison of PSA and $HPSA_5$ before and after modification reveals peak values at 1734 and 3630 cm^{-1}, which correspond to the characteristic absorption bands of carbonyl and hydroxyl groups, respectively. This indicates that the modification of PSA with DHBA leads to the formation of acetal structures, which in turn increases the characteristic absorption peak of carbonyl stretching vibrations as well as significantly augments the absorption band of hydroxyl groups. Concurrently, the characteristic absorption peak of double bond stretching vibrations at 1620 cm^{-1} is essentially eliminated, and no notable absorption peaks of double bond out-of-plane bending vibrations are observed at 990 cm^{-1}. This suggests that the emulsion polymerization reaction of acrylic monomers (such as BA, MMA, AA, HEA, and EHA) is relatively complete.

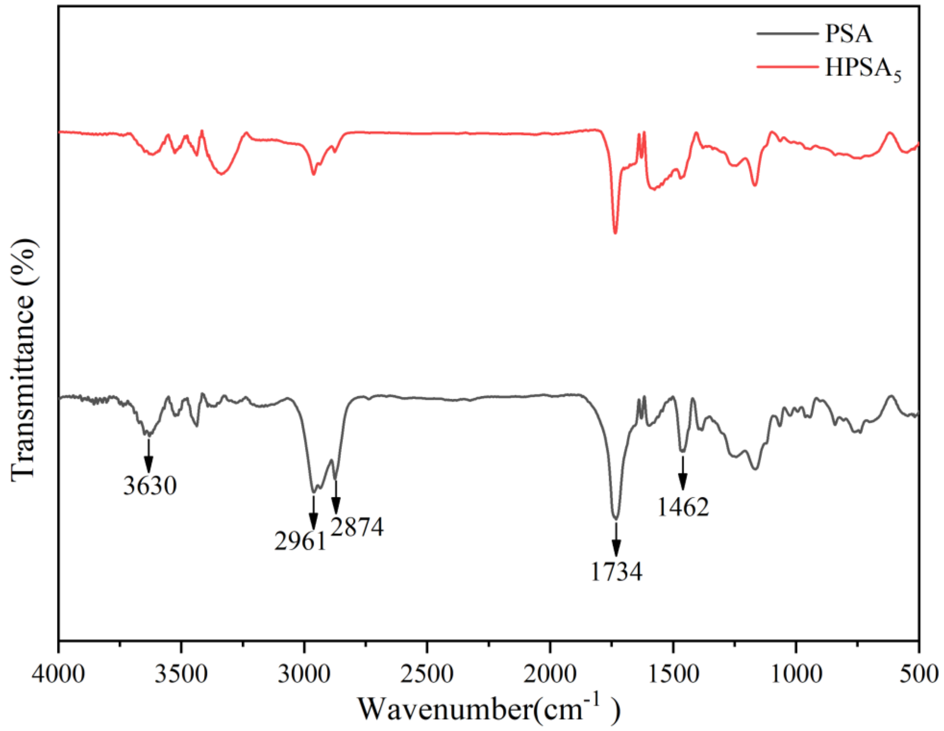

Figure S1. Latex film FTIR: PSA is modified acrylate colloid; $HPSA_5$ is added 5% DHBA.

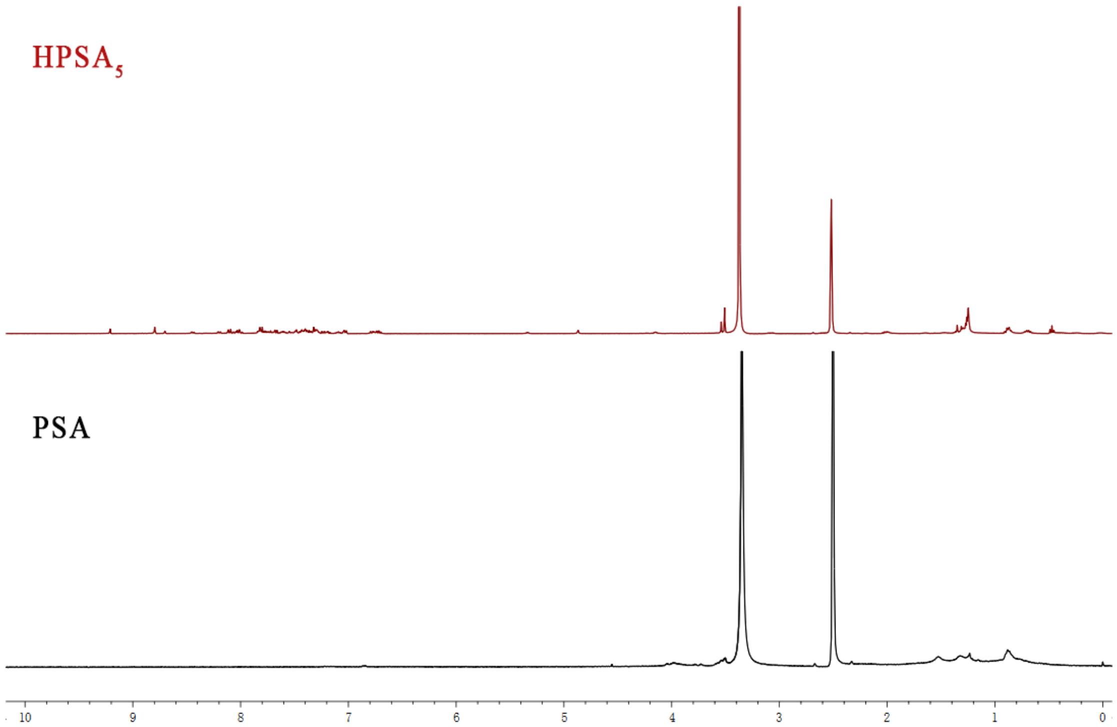

Figure S2. ^{1}H NMR spectrum of PSA and HPSAs.

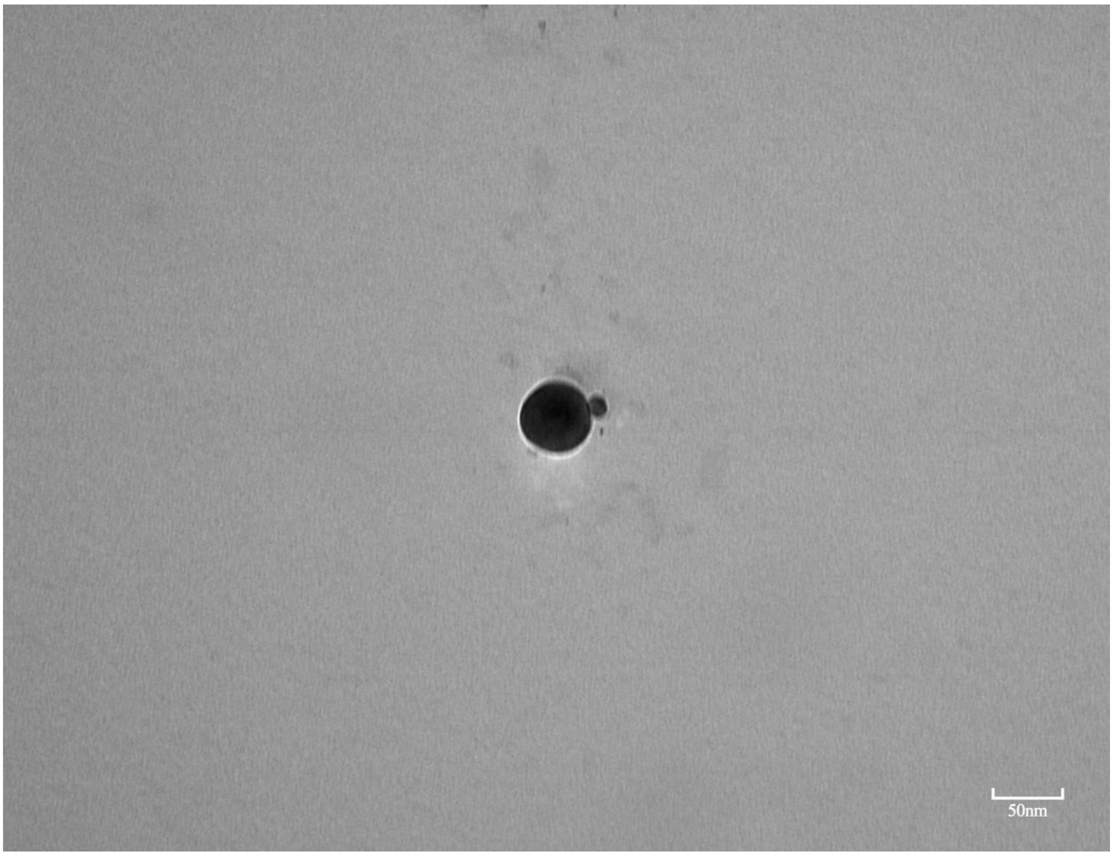

Figure S3. TEM projection images of $HPSA_5$.

Video V1. The damp heat shock test results of PSA and HPSAs
